# Skipping Breakfast and Lunch, as Well as Reducing Milk and Dairy Intake, Is Associated with Depressive Symptoms in Pregnant Adolescents

**DOI:** 10.3390/nu18040704

**Published:** 2026-02-22

**Authors:** Reyna Sámano, Estefania Aguirre-Minutti, Hugo Martínez-Rojano, Gabriela Chico-Barba, Ricardo Gamboa, Carmen Hernández-Chávez, María Eugenia Mendoza-Flores, Erika González-Medina, Primavera Pérez-Romero

**Affiliations:** 1 Coordinación de Nutrición y Bioprogramación, Instituto Nacional de Perinatología, Secretaría de Salud, Montes Urales 800, Lomas de Virreyes, Alcaldía Miguel Hidalgo, Mexico City 11000, Mexico; minuttiestefania@gmail.com (E.A.-M.); gabyc3@gmail.com (G.C.-B.); tina14mx@yahoo.com (M.E.M.-F.); 2 Escuela de Dietética y Nutrición del ISSSTE, Callejón Vía, Av. San Fernando No. 12, San Pedro Apóstol, Tlalpan, Mexico City 14070, Mexicoprimavera.perez@ednissste.com.mx (P.P.-R.); 3 Sección de Posgrado e Investigación de la Escuela Superior de Medicina del Instituto Politécnico Nacional, Plan de San Luis y Díaz Mirón s/n, Colonia Casco de Santo Tómas, Alcaldía Miguel Hidalgo, Mexico City 11340, Mexico; 4 Departamento de Fisiología, Instituto Nacional de Cardiología “Ignacio Chávez”, Mexico City 14080, Mexico; rgamboaa_2000@yahoo.com; 5 Departamento de Neurobiología del Desarrollo, Instituto Nacional de Perinatología, Secretaría de Salud, Montes Urales 800, Lomas de Virreyes, Alcaldía Miguel Hidalgo, Mexico City 11000, Mexico; karmenhdez@hotmail.com

**Keywords:** depression, depressive symptoms, adolescent pregnancy, eating habits, mental health, skipping breakfast

## Abstract

Background and objective: Depression is the most common mental health problem in women during pregnancy, associated with psychological, social, and medical factors characteristic of this stage. However, a lack of knowledge and limited attention to this condition can aggravate its consequences and restrict access to appropriate treatment. This research seeks to fill a gap in the scientific literature by exploring the association between eating habits and dietary diversity with depressive symptomatology in a group with high psychosocial vulnerability: pregnant adolescents. Material and methods: A cross-sectional analytical study was conducted with a sample of 344 pregnant adolescents attending prenatal care at the National Institute of Perinatology (INPer), a tertiary care center. Non-probabilistic sampling was used for recruitment. Relevant information was collected using a pre-validated structured questionnaire administered via interview. Depressive symptomatology was assessed using the Edinburgh Postnatal Depression Scale (EPDS), with a score of ≥12 considered indicative of a higher risk of depression. Eating habits were evaluated based on meal omission, activities performed during meals, and dietary diversity, comparing them with national recommendations. Food group consumption was assessed using a semi-quantitative Food Frequency Questionnaire (FFQ). Robust variance Poisson regression models were employed to evaluate the independent association between undesirable eating habits, inadequate food group intake, and the presence of depressive symptomatology. Results: A significant association was observed between the presence of depressive symptoms (EPDS ≥ 12) and the omission of main meals. Specifically, skipping breakfast was associated with a higher prevalence of EPDS scores ≥ 12 (aPR = 1.55; 95% CI: 1.10–2.19; *p* = 0.013). Similarly, adolescents who skipped lunch showed a higher prevalence of depressive symptomatology compared to those who did not (aPR = 2.02; 95% CI: 1.11–3.68; *p* = 0.022). Regarding food groups, only insufficient intake of milk and dairy products was significantly associated with the presence of depressive symptoms (aPR: 1.78; 95% CI: 1.16–2.73; *p* = 0.008). Conclusions: This cross-sectional study found a significant association between breakfast skipping, distraction while eating, and inadequate dairy intake with a higher prevalence of depressive symptoms in socially vulnerable pregnant adolescents treated at a tertiary care center. However, due to the study’s design, causality or the direction of the relationship cannot be established (it could be bidirectional), and it cannot be affirmed that modifying the diet will necessarily reduce depression. Furthermore, the results are not generalizable to all pregnant adolescents, and future research (longitudinal or interventional) is needed to better understand these associations before developing specific dietary interventions.

## 1. Introduction

Adolescence is a critical stage of human development, marked by significant emotional, physical, cultural, and social transitions that can modulate key behaviors, including eating habits and nutritional intake [1]. There is a growing body of scientific evidence suggesting that dietary patterns play an essential role in mental health, influencing the manifestation of disorders such as anxiety and depression [2]. Consequently, interest has increased in examining dietary factors as potentially modifiable risk elements for psychological well-being in adolescents.

Furthermore, depression stands as the most prevalent mental disorder during pregnancy and one of the leading causes of disability in women globally [3]. This condition, which can frequently progress to postpartum depression, significantly increases maternal morbidity and mortality if timely treatment is not received [4]. Clinically, a depressive mood is associated with a deficit in maternal self-care, manifested in inadequate nutrition, substance use, and poor adherence to prenatal care [5,6,7]. These behaviors not only affect the mother but also compromise fetal development, increasing the risk of low birth weight and preterm births [5,6]. The repercussions extend into childhood, where children of mothers with depression present a higher risk of malnutrition, sleep disorders, breastfeeding difficulties, and alterations in cognitive development and attachment [5,8,9,10]. Additionally, this pathology imposes a substantial economic burden on both developed and developing nations [11], underscoring the imperative need to identify modifiable risk factors [12].

Given the urgency of identifying these modifiable risk factors and considering the growing relevance of nutrition as a key determinant in mental health [13,14], along with the high prevalence of depression in the second trimester of gestation in this population [15], the purpose of this study was to determine the association between eating habits and dietary diversity with depressive symptomatology in pregnant adolescents treated at the National Institute of Perinatology.

## 2. Materials and Methods

A cross-sectional study was conducted with pregnant adolescents attending prenatal care at the National Institute of Perinatology (INPer) “Isidro Espinosa de los Reyes” in Mexico City. INPer is a tertiary hospital that provides care for adolescent pregnancies, which are considered high-risk. It should be noted that, although the vast majority of the adolescents treated do not present other comorbidities, their care at INPer is justified by their status as pregnant adolescents from low or very low socioeconomic levels, without the possibility of accessing private healthcare facilities.

### 2.1. Participant Recruitment

All pregnant adolescents attending the outpatient clinic of INPer were invited to participate in the study. Of the 377 invited adolescents, 344 met the selection criteria and agreed to participate. Participant recruitment was conducted between January 2020 and July 2025. All participants signed a written informed consent form, along with the corresponding assent from their parents or legal guardians, as applicable.

### 2.2. Participant Selection Criteria

Inclusion criteria were: pregnant adolescents aged between 10 and 19 years; primigravidae; singleton and intrauterine pregnancy; no prior diagnosis of metabolic, autoimmune, infectious, cardiovascular, or endocrine diseases. No restriction was applied regarding pre-pregnancy Body Mass Index (pBMI).

The exclusion of these adolescents was performed with the aim of homogenizing the sample and reducing the risk of confounding bias. Medical and pharmacological conditions, as well as traumatic psychosocial situations, which are major determinants of depression or metabolically alter food intake, were intentionally excluded. This strategy enhances the study’s internal validity, ensuring that the observed associations between eating habits and depressive symptomatology were not mediated or masked by underlying diseases, medication side effects, or severe prior traumas.

However, this specific exclusion, particularly that of adolescents with a previous diagnosis or treatment for psychiatric disorders (especially depression), implies an important consideration. By removing individuals with a history of depression from the sample, the study may have downwardly biased the observed prevalence and severity of depressive symptomatology. Consequently, the results obtained with the EPDS reflect associations within a subpopulation of pregnant adolescents without a prior psychiatric history. This is crucial for interpretability, as factors associated with the onset of depressive symptoms in individuals without a history may differ from those involved in the recurrence or exacerbation of depression in those who do have a history. Therefore, the results regarding the association between eating habits and depressive symptoms may be more applicable to new-onset cases or less severe symptomatology, limiting generalizability to the total population of pregnant adolescents, which would include those with a more complex psychiatric history.

### 2.3. Data Collection

Data were collected using a structured and previously validated questionnaire, administered through personal interviews. Data collection was carried out by a physician, a nurse, and two trained nutritionists, all affiliated with the Coordination of Nutrition and Bioprogramming of the National Institute of Perinatology, Ministry of Health of Mexico.

The questionnaire addressed various dimensions. Sociodemographic data (e.g., age, educational level (primary, secondary, high school), marital status, and socioeconomic status) were collected, and lifestyle and health habits (e.g., breakfast frequency, physical activity level, sleep duration and quality) were assessed. Eating habits and diversity were recorded through a food frequency questionnaire, supplemented with additional structured questions on the frequency of meal skipping, the number of complete meals per day, missed mealtimes, location and company during meals, and activities performed while eating.

For most cases, gestational age (GA) confirmation was performed using the estimated date of delivery obtained by ultrasound. When medical records were unavailable, GA was determined based on self-reported information.

Socioeconomic status was assessed using a validated questionnaire that was based on the methodology of the Mexican Association of Market Research and Public Opinion Agencies (AMAI). This questionnaire comprises eight questions addressing family assets, recreational activities, contracted services, and other relevant factors. The responses allow for the assignment of the adolescent’s family to one of six socioeconomic status categories: A/B (the highest standard of living), C+ (above average), C (average), D+ (below average), D (low or austere), and E (the lowest income and standard of living) [16].

Civil status was classified, following an approach that reflects both legal formality and cohabitation status, into two main categories with their respective subcategories:

1. In Union:

Married/Legally United: Includes adolescents who have legally married or who live in a legally recognized union.

Consensual/Free Union (Cohabiting): Includes adolescents who live with a partner in a stable and public manner, acting as if they were married, but without having legally formalized the union.

2. Not in Union:

Single (Never Married/Never in Union): Adolescents who have never married nor lived in a consensual union.

Separated: Adolescents who, having been married or in a consensual union, no longer live with their partner, even if the legal or social bond is not formally dissolved.

Divorced: Adolescents whose marriage has been legally dissolved.

Widowed: Adolescents whose spouse or partner has passed away.

For statistical analysis purposes, the categories ‘Separated,’ ‘Divorced,’ and ‘Widowed’ are often grouped with ‘Single’ into a general ‘Not in Union’ category, due to their low individual prevalence and their current status of not cohabiting with a partner.

### 2.4. Assessment of Depression Symptoms

The Edinburgh Postnatal Depression Scale (EPDS) is a rapid questionnaire that takes approximately five minutes to administer. It contains 10 questions that assess symptoms experienced during the previous week. Each question is scored from 0 to 3, with a total score ranging from 0 to 30. An EPDS score of 12 or more was used as a threshold to suggest a high risk of depression, given its validation for antenatal screening. This threshold is effective, correctly identifying 86% of cases (sensitivity) and correctly classifying negative cases 78% of the time (specificity) [17,18]. The internal consistency of the scale in our sample was also evaluated (Cronbach’s alpha = 0.826), indicating good reliability. All pregnant adolescents who scored 12 or higher on this scale were attended by the Psychology staff at INPer, who provided them with follow-up and care in the outpatient clinic.

### 2.5. Dietary Intake and Eating Habits

Food group consumption was assessed to characterize dietary intake using a semi-quantitative food frequency questionnaire (FFQ) [19]. The intake of nine food groups was measured. The dietary guidelines for the Mexican population were used as the reference standard. These guidelines present the following food groups and their recommended portions: vegetables (>3); fruits (3–5); legumes (2–2.5); cereals and grains (8–11); meat, cheese, and eggs (hereinafter “animal-source foods”) (3.5–4); fats and oils (3–5); milk and yogurt (2–2.5); table sugar (<5); and sugar-sweetened beverages (0) [20].

Participants reported their frequency of consumption over the preceding trimesters. Given that macronutrient intake is known to remain relatively stable during pregnancy [21], a measurement was obtained in the second or third trimester. Interviewers used food replicas, as well as standard measuring cups, spoons, and glasses, to improve portion size estimation. Subsequently, the number of portions consumed was compared with the recommendations for the Mexican population [20]. The number of portions for each food group used as a reference is listed in Supplementary Table S1. Consumption was considered adequate when the recommended number of portions was met. Inadequate consumption (excessive or insufficient) was defined as when participants consumed more or fewer portions than the recommended range.

Additionally, participants were asked about the following eating habits: number of meals; frequency of skipping meals (never, 1–3 times, 4–5 times per week); with whom they ate (alone, with family, or with friends); where they ate (away from home or at home); and what activities they performed while eating (doing housework, watching television or using a cell phone, or simply eating).

To ensure confidentiality, all survey responses, dietary recalls, and food frequency questionnaire data were recorded using anonymous study identifiers.

### 2.6. Ethical Considerations

This research was classified as minimum risk, as established in Article 17 of the General Health Law on Health Research [22,23]. This classification was made because it did not involve invasive interventions and prioritized confidentiality, despite addressing sensitive mental health topics. Before data collection, pregnant adolescents who met the selection criteria were invited to participate. Those who accepted were informed in detail about the study and provided their informed assent. Additionally, their parents or guardians provided the corresponding informed consent, ensuring a complete understanding of the research process. The research protocols (registration numbers 2017-2-101 and 2024-1-61) were approved by the institutional Research, Ethics, and Biosafety Committees of the National Institute of Perinatology Isidro Espinosa de los Reyes.

### 2.7. Statistical Analysis

To characterize the sample, a comprehensive descriptive analysis was performed. Qualitative variables were summarized using frequencies and percentages. For continuous quantitative variables, the normality of their distribution was assessed with the Kolmogorov–Smirnov test. Variables showing a normal distribution were presented as mean ± standard deviation, while variables without a normal distribution were expressed as median [interquartile range].

Subsequently, to investigate the relationship between undesirable eating habits, inadequate food consumption, and the presence of depressive symptomatology, Pearson’s chi-square test (two-tailed) was applied. For variables with low cell counts (or low expected frequencies), Fisher’s exact test (two-tailed) was used. These tests allowed for the comparison of the frequency of these factors between the groups with and without depressive symptomatology, identifying significant differences in proportions.

To evaluate the independent association between undesirable eating habits (or “unhealthy dietary patterns”), inadequate intake of food groups, and the presence of depressive symptomatology, robust variance Poisson regression models were employed. Depressive symptomatology was considered the dependent variable. Each model included potential confounding factors: age, socioeconomic status (SES), occupation, marital status, educational level, educational lag, pBMI, morning sleep duration, and physical activity. The selection of these covariates was based on their recognized associations with depressive symptomatology in the literature, which allowed for the adjustment of these variables and provided more precise estimates of the effect of each dietary behavior. [24,25,26].

The following behavioral indicators of eating habits were examined: skipping meals (breakfast, lunch, dinner, and snacks), eating out, eating alone, and eating while using screens. These variables, which reflect the participants’ actual eating behaviors, were analyzed as independent exposures in separate robust variance Poisson regression models. Furthermore, the consumption of multiple food groups (vegetables, fruits, legumes, animal-source foods, dairy, cereals, fats, and sugary drinks) was used as an indirect measure of diet quality. Each of these food groups was analyzed as an independent exposure in separate Poisson regression models.

All statistical analyses were carried out using the statistical package SPSS, version 19 (IBM SPSS Inc., Chicago, IL, USA). A statistical significance level of *p* ≤ 0.05 was set for all two-tailed tests.

## 3. Results

Of the 377 pregnant adolescents who agreed to participate, only 344 met the inclusion criteria. The main reasons for exclusion were incomplete questionnaires and missing data in the assessment scales. Overall, 37.2% of the sample exhibited depressive symptoms (EPDS score ≥ 12).

Table 1 shows that below-average socioeconomic status and being married/cohabiting were more frequent among adolescents with depressive symptoms (EPDS score ≥ 12). The other variables did not show statistically significant differences. Only 12% of the total sample had completed secondary education, and 80% reported that household chores were their main occupation.

In Table 2, it was observed that most participants were in the second trimester of pregnancy (53%). Although no statistically significant differences were noted between the groups for these variables, a considerable frequency of both insufficient (37%) and excessive (36%) gestational weight gain was identified. Regarding birth weight, babies with adequate weight for gestational age predominated; however, 21% were classified as small for gestational age.

A higher frequency of pregnant adolescents with depressive symptoms was observed among those who skipped breakfast (92% vs. 79% who did not skip it, *p* = 0.001) or ate while using a screen (67% vs. 55%, *p* = 0.032), compared to participants who did not exhibit these behaviors.

Among all participants, the most common unhealthy eating behaviors (present in more than 50% of them) were eating while using screens (cell phones, laptops, tablets, or television), meal skipping ≥3 times per week, breakfast skipping, and snack skipping (Supplementary Table S2).

Table 3 presents the frequency distribution of the consumption of various food groups, and a significant difference was observed in the frequency of dairy and dairy product consumption, where inadequate consumption was considerably more common in the group with depressive symptomatology (84% vs. 70%). Although tendencies towards a higher inadequate intake of fats (*p* = 0.078) and sugars (*p* = 0.052) were identified in the group with depressive symptomatology, these differences did not reach statistical significance. It is worth noting that, in general, less than 50% of participants reported adequate consumption of vegetables, fruits, legumes, cereals, and foods of animal origin.

Skipping breakfast was associated with an EPDS score above the cutoff of 12 (aPR = 1.55; 95% CI: 1.10–2.19; *p* = 0.013). Likewise, adolescents who skipped lunch showed a higher prevalence of an EPDS score above the cutoff of 12, compared to those who did not skip lunch (aPR = 2.02; 95% CI: 1.11–3.68; *p* = 0.022). Engaging in other activities while eating also showed an association with an EPDS score above the cutoff of 12 (aPR = 1.39; 95% CI: 1.02–1.95; *p* = 0.049). Eating alone showed a trend towards a higher prevalence of depressive symptoms; however, the association did not reach statistical significance (aPR = 1.37; 95% CI: 0.97–1.95; *p* = 0.077) (see Table 4).

Results in Table 5 indicate that, among the different food groups analyzed, only insufficient intake of milk and dairy products was associated with the presence of depressive symptomatology (aPR: 1.78 95% CI 1.16–2.73, *p* = 0.008) in the studied adolescent group.

## 4. Discussion

Our research revealed an association between certain dietary habits and the presence of depressive symptomatology. Specifically, habits such as skipping breakfast or lunch were associated with a higher score on the EPDS. Furthermore, when analyzing dietary diversity, we observed that low consumption of milk and dairy products was significantly associated with the presence of depressive symptomatology. These findings suggest that dietary patterns and quality could influence the mental health of pregnant adolescents.

### 4.1. Eating Habits

#### 4.1.1. Skipping Breakfast

Breakfast skipping in pregnant adolescents was associated with an EPDS score ≥ 12, suggesting a higher likelihood of depressive symptoms. Furthermore, our results align with existing literature identifying unhealthy dietary habits and lifestyles characteristic of adolescents who skip breakfast [27,28,29,30,31,32,33]. However, our findings are subject to methodological limitations, limitations in external validity, and the possibility of reverse causality.

Furthermore, there is evidence linking prolonged skipping of breakfast with an increased risk of depression, suggesting an influence on mental well-being [34,35]. Consequently, scientific data highlight the relevance of breakfast as a factor contributing to mood and the prevention of depressive states.

Although the precise mechanisms by which breakfast exerts a beneficial effect on depressive symptoms are not fully elucidated, the observed inverse relationship can be attributed to several plausible explanations that influence the manifestation of depression. A prominent hypothesis points to the role of carbohydrate intake, which is higher in those who regularly consume breakfast [36]. After an overnight fast, the body needs energy; inadequate replenishment of this energy can negatively affect mood, which is associated with the development of depression [37,38,39,40].

Nutrient intake could contribute to explaining the relationship between breakfast frequency and depressive symptoms. Previous studies suggest that breakfast skippers consume fewer micronutrients whose adequate intake has been associated with a lower risk of presenting depressive symptoms [41,42,43,44]. However, our results did not show a conclusive association between the consumption of these elements and said symptoms, suggesting the need for further research. Likewise, factors such as socioeconomic status and daily habits could influence the relationship between breakfast skipping and the presence of depressive symptoms.

Additionally, daily behavioral patterns could act as mediators in the link between breakfast omission and susceptibility to depressive symptoms. Existing literature indicates that individuals who skip breakfast often concurrently exhibit other health behaviors, such as problematic internet use [45], sedentary behavior, and an evening chronotype [46,47]. It is relevant to highlight that a relationship has been identified between these factors—internet addiction, low physical activity, and a preference for evening schedules—and an increased risk of developing depression.

Despite the complexity and discrepancies existing in the general literature regarding this association, data from prospective studies are scarce and not always conclusive [48]. Certain investigations point to a potential benefit; however, their findings are restricted by methodological limitations and issues of external validity, while other studies have failed to establish a significant correlation [49]. In this regard, the results of our study align with the majority of investigations that have demonstrated an association between breakfast omission and an increased risk of depressive symptoms in adolescents.

#### 4.1.2. Activities Performed While Eating

Regarding the practice of engaging in other activities during food intake, our results align with the findings of a study conducted in adolescents aged 11 to 18 in Colombia [50]. This research, which utilized the Pediatric Quality of Life Inventory questionnaire, reported that participants who watched television for ≥2 h per day exhibited lower quality of life scores, indicating greater impairment in emotional and psychological dimensions, as well as in relational capacity and school performance.

Although this study does not directly address the relationship between watching television while eating and depressive symptoms, it suggests that excessive screen exposure can negatively impact the emotional well-being of young people. On the other hand, a study with Australian adolescents [51] observed that those who watched television while eating showed alterations in hunger and satiety sensitivity, resulting in a greater food intake compared to those who ate without distractions. Likewise, research conducted in Colombia with children aged 9 to 12 [52] associated daily television exposure of 2 or more hours with overweight and obesity, a phenomenon likely linked to increased consumption of ultra-processed products.

Collectively, this evidence suggests that eating while engaging in simultaneous activities, such as watching television, can have negative consequences for the physical and mental health of pregnant adolescents, promoting unhealthy eating habits that, ultimately, can affect emotional well-being. It is important to recognize that, given the observational nature of these studies, these associations could be bidirectional; for example, impaired emotional well-being could also influence the propensity to seek distractions during meals, creating a complex cycle.

### 4.2. Dietary Diversity

Furthermore, various modifiable lifestyle factors influence the etiology of common mental disorders, with nutrition standing out as an area of growing scientific interest. In this context, dietary diversity constitutes a key indicator, defined as the variety of food groups consumed over a specific period. A varied diet is fundamental for ensuring adequate nutrition, as it provides a broad spectrum of essential macronutrients and micronutrients. Given that inadequate dietary patterns correlate with an increased risk of malnutrition and chronic diseases such as depression, international and local guidelines recommend optimizing dietary diversity [53]. A diverse diet should include, in varying proportions, vegetables, fruits, legumes and their derivatives, nuts, meat, eggs, fish, dairy products and their derivatives, tea, cereals, oils or fats [54].

Likewise, our study demonstrated that inadequate consumption of dairy and fats is associated with a higher risk of depressive symptoms in pregnant adolescents. These findings are consistent with those reported by Poorrezaeian et al. [55] in a study conducted with 360 women in Tehran, which highlighted the importance of dietary diversity and the antioxidant potential of certain foods, especially milk and its derivatives. Furthermore, they align with the work of Chan et al. [56], who observed that a reduction in the consumption of dairy products, vegetables, and fruits was associated with a higher probability of experiencing depressive symptoms during pregnancy and the puerperium in a group of 300 women in Taiwan.

While our study is observational in nature and does not explore the underlying physiological mechanisms of these associations, which implies that direct causality or the direction of the relationship cannot be established, the scientific literature suggests various pathways through which nutrition could influence mental health. It is important to consider that this association could be bidirectional, where mental health status also impacts dietary choices and habits. For example, it has been proposed that oxidative stress could be involved in mental disorders, and that the consumption of foods rich in vitamins, carotenoids, and polyphenols, such as fruits and vegetables, could be beneficial by providing a significant antioxidant contribution [57,58,59]. Additionally, the presence of riboflavin in dairy products, along with the flavonoids and tocopherols found in nuts, suggests that these products are sources of compounds that could help counteract such oxidative stress [55]. Moreover, it has been suggested that inadequate nutrition could be a contributing factor to the etiology of depressive symptoms, as it is hypothesized to promote homocysteine accumulation and decrease brain monoamine synthesis [55,60]. Finally, diet influences the concentrations of eicosapentaenoic acid (EPA) and docosahexaenoic acid (DHA); evidence from a meta-analysis indicates that individuals with depressive symptoms exhibit significantly reduced levels of these fatty acids [61].

### 4.3. Strengths and Limitations of the Study

The main strength of this research lies in its comprehensive approach to analyzing dietary patterns. Unlike other studies that often focus exclusively on the frequency of food group consumption, the present work examined key behavioral habits such as meal skipping, social interaction during meals, and participation in simultaneous activities. This approach provides a more detailed understanding of the diverse eating habits of pregnant adolescents and, thanks to the methodological design used, allows for the exploration of a possible association with depressive symptomatology.

However, the present study presents certain limitations that must be acknowledged. Firstly, its cross-sectional observational design prevents establishing direct causal relationships or determining the directionality of the associations; although an association is observed, this could be inverse, meaning that depressive symptomatology could be influencing or altering dietary choices and habits. To establish the unidirectionality of these associations, future research with longitudinal or interventional designs is crucial.

Secondly, numerous psychosocial factors not measured in this study could act as common determinants of both depressive symptomatology and eating habits. Aspects such as chronic stress, lack of social or family support, intrafamilial violence, economic insecurity, limited access to nutritious food, or a history of trauma, can simultaneously influence mood and dietary decisions (for example, through emotional eating or meal omission due to limited resources or lack of time). The exhaustive non-inclusion of these variables as potential residual confounding factors limits our ability to attribute the observed association solely to the direct relationship between diet and depressive symptoms. It is plausible that one or several of these underlying psychosocial variables are mediating or modifying the observed relationship.

Thirdly, it should be noted that, while the Edinburgh Postnatal Depression Scale (EPDS) is a validated screening tool for depressive symptomatology, it lacks the diagnostic precision of a clinical psychiatric interview, considered the “gold standard” for the diagnosis of depression.

Finally, the sample was limited to pregnant adolescents from low or modest socioeconomic strata, without access to private medical services, attended at a single tertiary medical center in Mexico City. It is important to highlight that the care for these patients at the center is based on adolescent pregnancy as the main risk factor, rather than on the presence of additional comorbidities. This specificity limits the external validity and generalizability of our findings to populations with different sociodemographic characteristics or from other countries or regions.

### 4.4. Clinical Implications

The findings of our observational study reinforce the importance of promoting dietary diversity and healthy eating habits during pregnancy in vulnerable groups, such as pregnant adolescents. While we cannot establish a direct causal relationship, the observed association between these factors and a lower risk of depressive symptomatology suggests that optimizing nutrition could be a valuable strategy for emotional well-being. In particular, adequate intake of dairy products (milk and derivatives) is relevant, given their known role in mood regulation and brain function. Furthermore, avoiding skipping breakfast and reducing distractions, while eating can help improve the perception of hunger and satiety cues, promoting more balanced and nutritious eating practices. In clinical practice, it is crucial to recognize the possible bidirectionality of these relationships, where mental health status can also influence dietary habits.

### 4.5. Future Perspectives

Longitudinal or intervention studies are needed to confirm the directionality and causality of the association between diet and depressive symptomatology in pregnant adolescents. Furthermore, it is essential to explore this relationship across different socioeconomic strata to understand the impact of social inequalities. Additionally, other sociodemographic factors linked to dietary habits and mental health should be considered, such as educational level, marital status, social support, and access to health services. The integration of these variables will enable the design of effective interventions that promote healthy eating and improve the mental health of these adolescents, addressing their specific needs.

## 5. Conclusions

This cross-sectional study analyzed the relationship between eating patterns and depressive symptomatology in pregnant adolescents with high psychosocial vulnerability attending a tertiary care center. A significant association was observed between breakfast skipping, distraction during food intake, and inadequate dairy product intake with a higher presence of depressive symptoms.

These findings suggest that such eating behaviors are not isolated events but rather coexist with alterations in maternal mental health. However, given the limitations of the cross-sectional design, it is not possible to establish causality or direction. A bidirectional relationship or inverse causality is plausible, where depressive symptomatology itself could modulate eating behaviors and appetite. Therefore, it cannot be concluded from these data that dietary modification will necessarily result in a reduction in depressive symptoms.

It is important to note that, since the sample comes from a specialized tertiary institution and presents specific vulnerability characteristics, these results should not be generalized to the entire population of pregnant adolescents. Further longitudinal or intervention research is required to clarify the direction of these associations before specific dietary strategies aimed at mental health can be proposed for this population.

## Figures and Tables

**Table 1:** Sociodemographic characteristics of participants according to depressive symptoms (EPDS score ≥ 12).

Variables		All *n* = 344	With Depressive Symptoms *n* = 129, *n* (%)	Without Depressive Symptoms *n* = 215 *n* (%)	*p* *
Age (years) **		16 (15–17)	16 (15–17)	16 (15–17)	0.458
Education					
	Primary	87 (25)	34 (26)	53 (25)	0.279
	Secondary	215 (63)	75 (58)	140 (65)	
	High school	42 (12)	20 (15)	22 (10)	
Occupation					
	Housewife	276 (80)	104 (81)	172 (80)	0.890
	Study/work	68 (20)	25 (19)	43 (20)	
Civil status					
	Single	258 (75)	90 (70)	168 (78)	0.006
	Cohabiting/married	86 (25)	39 (30)	47 (22)	
Socioeconomic level					
	Below average	212 (62)	71 (55)	141 (66)	0.034
	Low or austere	132 (38)	58 (45)	74 (34)	
Family structure					
	Nuclear	184 (53)	64 (50)	120 (56)	0.428
	Extended	91 (27)	39 (30)	52 (24)	
	Uniparental	69 (20)	26 (20)	43 (20)	

* *p*-value by Square Chi Pearson; ** *p*-value by Mann–Whitney U test.

**Table 2:** Clinical characteristics by depressive symptom status (EPDS score ≥ 12).

Variable		All *n* = 344	With Depressive Symptoms *n* = 129, *n* (%)	Without Depressive Symptoms *n* = 215, *n* (%)	*p* *
Menarche age (years) ^a^		12 (11–12)	12 (11–12)	12 (11–12)	0.196 ^a^
	First trimester	26 (8)	7 (5)	19 (9)	0.425
	Second trimester	184 (53)	68 (53)	116 (54)	
	Third trimester	134 (39)	54 (42)	80 (37)	
Pregestational BMI					
	Normal	257 (75)	95 (74)	162 (75)	0.798
	Non-adequate ^b^	87 (25)	34 (26)	53 (25)	
Delivery					
	Vaginal	179 (52)	62 (35)	117 (65)	0.154
	Cesarean section	165 (48)	67 (41)	98 (59)	
Gestational age					
	Preterm	60 (17)	24 (19)	36 (17)	0.382
	Term	284 (83)	105 (81)	179 (83)	
Gestational weight gain					
	Insufficient	127 (37)	49 (38)	78 (36)	0.616
	Adequate	93 (27)	31 (24)	62 (29)	
	Excessive	124 (36)	49 (38)	75 (35)	
Birth-weight					
	Small for gestational age	72 (21)	27 (21)	45 (21)	0.986
	Adequate	265 (77)	99 (77)	166 (77)	
	Large for gestational age	7 (2)	3 (2)	4 (2)	

* *p*-values were calculated using Pearson’s chi-square test; ^a^ medians were compared using the Mann–Whitney U test; ^b^ non-adequate nutritional status includes underweight, overweight, and obesity; BMI: body mass index.

**Table 3:** Frequency of food group consumption by depressive symptom status (EPDS score ≥ 12).

Food Groups	Consumption (Servings)	Without Depressive Symptoms, *n* = 215, *n* (%)	With Depressive Symptoms, *n* = 129, *n* (%)	*p* *
Vegetables	Adequate > 3	33 (15)	14 (11)	0.261
	Inadequate < 3	182 (85)	115 (89)	
Fruits	Adequate 3–4	48 (22)	34 (26)	0.434
	Inadequate < 3	167 (78)	95 (74)	
Legumes	Adequate 2–2.5	32 (15)	16 (12)	0.630
	Inadequate < 2	183 (85)	113 (88)	
Cereals **	Adequate 8–11	17 (8)	9 (7)	0.835
	Inadequate < 8 or >11	198 (92)	120 (93)	
Foods of Animal Origin **	Adequate 3.5–4	7 (3)	3 (2)	0.749
	Inadequate < 3.5	208 (97)	126 (98)	
Milk and dairy products	Adequate 2–2.5	64 (30)	21 (16)	0.006
	Inadequate < 2	151 (70)	108 (84)	
Fats	Adequate 3–5	81 (38)	36 (28)	0.078
	Inadequate < 3 or >5	134 (62)	93 (72)	
Sugar-sweetened beverages	Adequate 0	45 (21)	18 (14)	0.115
	Inadequate 1 or +	170 (79)	111 (86)	
Sugars **	Adequate < 5	215 (100)	126 (98)	0.052
	Inadequate > 5	0 (0)	3 (2)	
Water	Adequate	22 (10)	14 (11)	0.857
	Inadequate	193 (90)	115 (89)	

* *p*-value by Pearson χ^2^ test; ** *p*-value by Fisher’s exact test.

**Table 4:** Association between non-recommended eating habits and depressive symptoms (EPDS score ≥ 12). Poisson regression model with robust variance.

Factors	aPR	95% CI	*p*	*Β*	95% CI
Skipping breakfast	1.55	1.10–2.19	0.013	0.439	0.092–0.785
Skipping lunch	2.02	1.11–3.68	0.022	0.702	0.101–1.304
Skipping dinner	1.50	0.77–2.90	0.231	0.404	−0.250–1.065
Skipping supper	1.06	0.71–1.58	0.790	0.055	−0.349–0.459
Breakfast away from home	1.19	0.82–1.72	0.364	0.171	−0.199–0.542
Lunch away from home	1.04	0.63–1.72	0.884	0.037	−0.469–0.544
Dinner away from home	0.94	0.63–1.41	0.760	−0.063	−0.468–0.342
Eating meals alone	1.37	0.97–1.95	0.077	0.316	−0.036–0.666
Eating while using screens	1.39	1.02–1.95	0.049	0.327	−0.012–0.666

aPR: Adjusted Prevalence Ratio. All models were adjusted for age, marital status, socioeconomic status, educational level, educational lag, pBMI, sleep hours, and physical activity.

**Table 5:** Association between dietary patterns and prevalence of depressive symptoms in pregnant adolescents (EPDS score ≥ 12). Poisson regression model with robust variance.

Factors	aPR	95% CI	*p*	*β*	95% CI
Insufficient intake of vegetables	1.55	0.83–2.88	0.170	0.436	−0.186–2.880
Excessive intake of cereals	1.10	0.52–2.34	0.802	0.096	−0.657–0.850
Inadequate intake of animal-based foods	1.47	0.88–2.45	0.143	0.384	−0.129–0.898
Low intake of legumes	1.28	0.41–4.00	0.243	0.243	−0.900–1.386
Low intake of milk and dairy products	1.78	1.16–2.73	0.008	0.577	0.151–1.003
Excessive intake of fats	1.39	0.93–2.08	0.112	0.327	−0.076–0.730
Excessive consumption of sugary drinks	1.23	0.68–2.10	0.534	0.179	−0.385–0.743
Low intake water	1.20	0.68–2.14	0.849	0.050	−0.465–0.565

aPR: Adjusted prevalence ratio. Models were adjusted for sociodemographic variables (age, marital status, socioeconomic status, education) and clinical and lifestyle variables (pBMI, physical activity, sleep duration).

## Data Availability

The data presented in this study are available from the corresponding authors upon reasonable request.

## Supplementary Materials

The following supporting information can be downloaded at: https://www.mdpi.com/article/10.3390/nu18040704/s1, Table S1: Recommended intake and number of servings of food groups for adolescent pregnancy; Table S2: Unhealthy eating behaviors among participants with and without depressive symptoms.

## Abbreviations

The following abbreviations are used in this manuscript:

BMI	Body mass index
EPDS	Edinburgh Postnatal Depression Scale
GA	Gestational Age
INPer	Instituto Nacional de Perinatología (National Institute of Perinatology)
OR	Odds ratio
aPR	Adjusted Prevalence Ratio
