# Supplementary material for: Skipping Breakfast and Lunch, as Well as Reducing Milk and Dairy Intake, Is Associated with Depressive Symptoms in Pregnant Adolescents
Source: Nutrients. 2026 Feb 22;18(4):704. doi: 10.3390/nu18040704 (PMC12943198; doi:10.3390/nu18040704)
Supplement: Supplementary file 1 [file nutrients-18-00704-s001.zip › nutrients-4133656-supplementary.pdf]

Supplementary Table S1. Recommended intake and number of servings of food groups for adolescent pregnancy

Food Group	Recommendation (servings)
Vegetables	>3 0
Fruits	3–4
Grain and cereals	8–11
Legumes	2–2.5
Animal-source foods	3.5–4
Fat and oils	3–5
Milk and yogurt	2–2.5
Sugar table	<5
Sugar sweetened beverages	0

Academia Nacional de Medicina 2015. México (Fernández-Gaxiola et al. 2015).

Supplementary Table S2. Unhealthy eating behaviors among participants with and without depressive symptoms

	Consumption	Without Depressive Symptoms, *n*=215, *n* (%)	With Depressive Symptoms, *n*=129, *n* (%)	**p*
Meals skipping ≥ 3 times/week	Yes	119 (55)	77 (60)	0.250
	No	96 (45)	52 (40)	
Having less than 3 meals	Yes	47 (22)	40 (31)	**0.040**
	No	168 (78)	89 (69)	
Breakfast skipping	Yes	22 (10)	24 (19)	0.022
	No	193 (90)	105 (81)	
In the morning snack skipping	Yes	169 (79)	119 (92)	**0.001**
	No	46 (21)	10 (8)	
Lunch skipping**	Yes	3 (1)	5 (4)	0.135
	No	212 (99)	124 (96)	
In the evening snack skipping	Yes	186 (87)	120 (93)	**0.043**
	No	29 (13)	9 (7)	
Dinner skipping	Yes	32 (15)	25 (19)	0.174
	No	183 (85)	104 (81)	
Breakfast away from home	Yes	32 (15)	24 (19)	0.224
	No	183 (85)	105 (81)	
Having lunch away from home	Yes	20 (9)	12 (9)	0.581
	No	195 (91)	117 (9)	
Having dinner away from home	Yes	40 (19)	26 (20)	0.413
	No	175 (81)	103 (80)	
Eating breakfast alone	Yes	30 (12)	25 (19)	0.120
	No	185 (86)	104 (81)	
Eating lunch alone**	Yes	12 (6)	12 (9)	0.138
	No	203 (94)	117 (91)	
Eating dinner alone	Yes	39 (18)	28 (22)	0.251
	No	176 (82)	101 (78)	
Eating while using screens	Yes	118 (55)	86 (67)	**0.020**
	No	97 (45)	43 (33)	

**p*-values were calculated using the Pearson chi-square test (two-sided); ** p-values were calculated using Fisher's exact test (two-sided).
